# Multiple sclerosis: experimental models and reality

**DOI:** 10.1007/s00401-016-1631-4

**Published:** 2016-10-20

**Authors:** Hans Lassmann, Monika Bradl

**Affiliations:** Center for Brain Research, Medical University of Vienna, Spitalgasse 4, 1090 Vienna, Austria

**Keywords:** Multiple Sclerosis, Experimental Autoimmune Encephalomyelitis, Multiple Sclerosis Patient, Progressive Multiple Sclerosis, Myelin Oligodendrocyte Glycoprotein

## Abstract

One of the most frequent statements, provided in different variations in the introduction of experimental studies on multiple sclerosis (MS), is that “Multiple sclerosis is a demyelinating autoimmune disease and experimental autoimmune encephalomyelitis (EAE) is a suitable model to study its pathogenesis”. However, so far, no single experimental model covers the entire spectrum of the clinical, pathological, or immunological features of the disease. Many different models are available, which proved to be highly useful for studying different aspects of inflammation, demyelination, remyelination, and neurodegeneration in the central nervous system. However, the relevance of results from such models for MS pathogenesis has to be critically validated. Current EAE models are mainly based on inflammation, induced by auto-reactive CD4^+^ T-cells, and these models reflect important aspects of MS. However, pathological data and results from clinical trials in MS indicate that CD8^+^ T-cells and B-lymphocytes may play an important role in propagating inflammation and tissue damage in established MS. Viral models may reflect key features of MS-like inflammatory demyelination, but are difficult to use due to their very complex pathogenesis, involving direct virus-induced and immune-mediated mechanisms. Furthermore, evidence for a role of viruses in MS pathogenesis is indirect and limited, and an MS-specific virus infection has not been identified so far. Toxic models are highly useful to unravel mechanisms of de- and remyelination, but do not reflect other important aspects of MS pathology and pathogenesis. For all these reasons, it is important to select the right experimental model to answer specific questions in MS research.

## Introduction: basic features of MS, which should be mirrored in experimental models

MS is a chronic inflammatory demyelinating disease of the central nervous system (CNS) [91]. In most patients, the disease starts with a phase of relapses and remissions, which may after 10 to 15 years convert into the progressive phase. Ten to fifteen percent of the patients miss the relapsing phase of the disease and develop primary progressive MS [100]. Progressive MS is generally seen in older patients than relapsing remitting MS, suggesting that age-related changes of the brain play some role for the slow and steady increase of neurological disability in this phase [141]. On pathological examination, inflammation consisting of T-cell and B-cell infiltrates is invariably present in the CNS and lesions of MS patients. This is the case not only in the early relapsing, but also in the progressive phase, at least as long as there is evidence for active demyelination and neurodegeneration [53]. The degree of lymphocytic inflammation is higher in early than in late disease stages. The lymphocytic inflammatory infiltrates are dominated by CD8^+^ T-cells with much lower contribution of CD4^+^ T-cells and B-cells [24, 53, 62]. Recent results of clinical trials show profound effects of anti-inflammatory therapies, which globally target T- and B-cells or B-cells alone [150, 152], while therapies that specifically address CD4^+^ T-cell-mediated inflammation show low or even no effect [145, 161]. Whether the effect of B-cell-directed therapies is due to a blockade of B-cell-mediated inflammation alone, to the reduction of antigen presentation for T-cells or to the elimination of Epstein Barr virus infected B-cells as a driver of the chronic inflammatory process is currently unresolved [61]. Overall, however, the data indicate that CD8^+^ T-cells and/or B-cells may play a more prominent role in disease pathogenesis, when the disease is already established. This, however, does not exclude that CD4^+^ T-cells are involved in triggering the inflammatory cascade at disease onset, as suggested by recent genetic association studies [72].

**Fig. 1:**
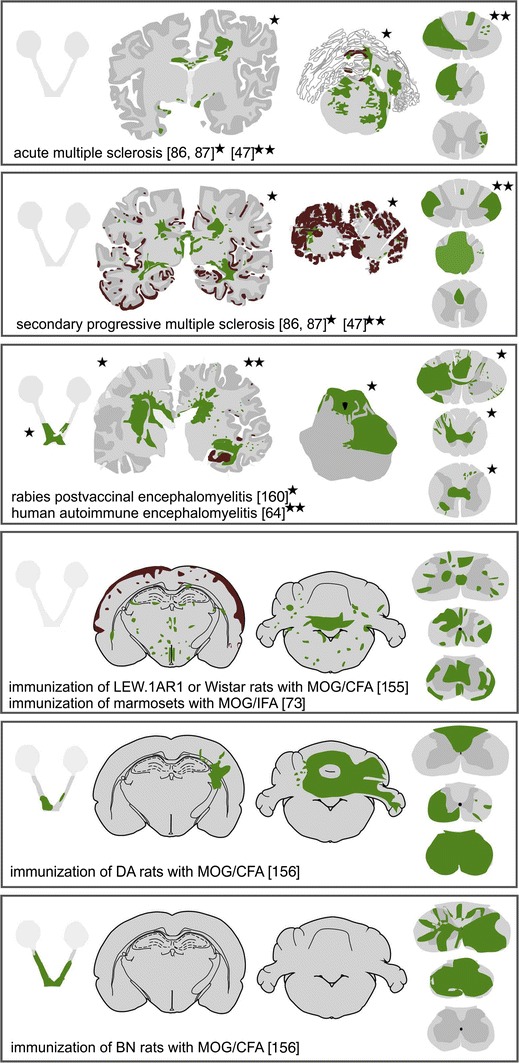

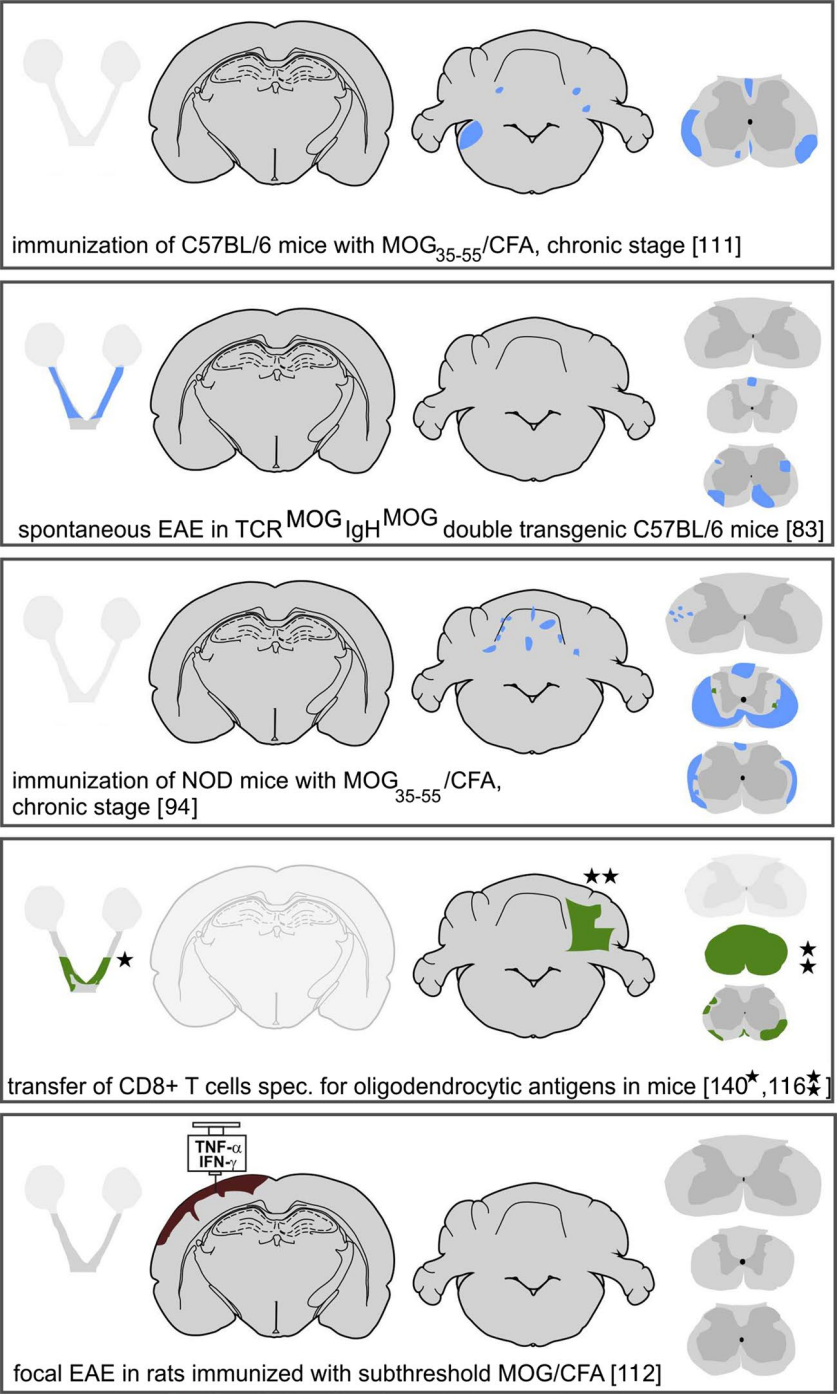
Distribution of demyelinating lesions in MS and different EAE-based models. The sites of demyelinated lesions were shown in camera lucida drawings of human brain sections, were projected into schemes redrawn after Paxinos and Watson [124] for rat and murine brain sections, or were outlined in optic nerve and spinal cord schemes. Areas of primary demyelination are shown in *green*, lesions with dominant axonal loss, and secondarily demyelinated areas in *blue*, and cortical demyelination in *brown*. *Shaded* schemes indicate lack of sufficient information for lesion distribution

The key feature distinguishing MS from other inflammatory diseases of the brain is the widespread primary demyelination, which gives rise to large focal lesions with complete myelin loss and partial sparing of axons (Fig. 1) [91]. This is most impressively seen in subpial demyelinated lesions in the cerebral cortex, which are absolutely specific for MS and were not seen in any other inflammatory condition of the brain [45, 113] (Fig. 1). In addition to demyelination, there is a partial and variable degeneration and loss of axons in the lesions [44, 81]. However, axonal degeneration, nerve cell loss, and dendritic/synaptic injury also occur in many other inflammatory conditions in the brain in the absence of primary demyelination and are, thus, not an MS-specific pathological feature. Active demyelination and neurodegeneration are associated with profound microglia activation and the presence of macrophage-like cells [45, 163]. In active lesions, astrocytes are activated and seem to be involved in the propagation of the inflammatory response and tissue injury [26]. In established chronic lesions, astrocytes form a dense glial scar.

Inflammation, primary demyelination, and neurodegeneration are the dominant features of both, relapsing and progressive MS (Fig. 1). So far, no qualitative difference in the pathology between these different disease stages has been unequivocally documented. However, the abundance of specific features differs quantitatively [57] and makes the following pathological alterations the most prominent features of progressive MS:

1. Slow expansion of pre-existing demyelinated lesions, while the formation of new white matter plaques with massive inflammation and blood brain barrier damage is sparse [52].
2. Progression of axonal degeneration in the plaques and in the normal appearing white matter, the latter in part due to retrograde and anterograde neuronal degeneration and in part related to inflammation in the meninges and the tissue [57]. This is seen in addition to the acute axonal injury in active MS lesions [53].
3. Presence of cortical (subpial) demyelinated lesions which are very prominent and may affect up to 90 % of the cortical ribbon [86] (Fig. 1).
4. Occurrence of all these changes on the background of a moderate lymphocytic inflammatory reaction associated with profound microglia activation in the lesions and the adjacent white matter [53].

### Is MS pathology reproduced in conditions of autoimmune encephalomyelitis in humans?

Before any experimental animal model of autoimmune encephalomyelitis has been developed, it was already clear that sensitization of humans with brain tissue or brain cells can be followed by neuroparalytic complications. This was seen as a side effect of anti-rabies vaccination using inactivated viruses insufficiently purified from brain cells or brain tissues which they have been propagated in. A large meta-analysis of data from 150,000 vaccinated individuals published between 1880 and 1928 revealed that neuroparalytic complications occurred in an incidence of about 1 out of 1000, and that affected patients most frequently presented with a clinical disease of acute polyradiculoneuritis or acute disseminated encephalomyelitis (ADEM; [157]). Only few of these patients developed a condition pathologically resembling MS [146, 160] (Fig. 1). We recently had the chance to study a similar case and found a pathological picture, which reflected all changes also seen in acute MS [64] (Fig. 1). The inflammatory infiltrates mainly consisted of B-lymphocytes and lower numbers of CD8^+^ T-cells and plasma cells, while CD4^+^ lymphocytes were virtually absent. These data clearly show that an MS-like disease can be induced by auto-sensitization with brain tissue, but there is a fundamental difference to MS. In all patients, disease subsided after the active sensitization process was stopped and there was no single patient, who developed chronic MS [157]. Similarly, in animal studies, it has been shown that the acute or chronic inflammatory demyelinating disease burns out when the peripheral brain antigen depot at the sensitization site has been removed [159]. This suggests that the propagation of chronic (autoimmune?) disease in MS requires stimulation by a persistent trigger within or outside the CNS.

### Models of experimental autoimmune encephalomyelitis

Based on the observation of an inflammatory, in part MS-like disease, after auto-sensitization in humans, attempts have been made to reproduce this disease in animal models [11, 135]. In the early phases of this research, immunization was performed with emulsions of brain tissue dissolved in saline. This resulted in a similar disease as described before in humans, albeit only in a fraction of the sensitized animals and after multiple vaccinations over a long time period. To improve reproducibility, strong adjuvants have been introduced into the sensitization protocol, in most cases complete Freund’s adjuvant, which provides a slow liberation of the sensitizing antigen from the inoculum and uses inactivated mycobacteria as a massive immune stimulant [75]. One effect of this adjuvant is that it massively stimulates phagocytic uptake and presentation of antigens and the subsequent activation and expansion of CD4^+^ T-lymphocytes [20]. Thus, nearly all of the models, which have so far been used, are biased towards a specific immune response mediated by MHC Class II restricted CD4^+^ T-lymphocytes. The disease induced by this sensitization process has been named experimental autoimmune encephalomyelitis (EAE), which is now one of the most widely used in vivo models to study immunology and brain inflammation. EAE can be induced in all vertebrates tested so far with a different degree of efficacy. Therefore, a widespread spectrum of models with different specific features is now available [1], most commonly using mice, rats, and primates (see below). The enthusiasm for the EAE models in MS research varied over time. In the early stages (1930–1960), the models have been seen as highly useful tools to study MS pathogenesis, but then, limitations became apparent, which indicated that such models are only useful for the investigation of very specific disease-related questions (1960–1990). The introduction of highly reproducible EAE in mice, using myelin oligodendrocyte glycoprotein MOG_35–55_ peptide for sensitization [111], offered to address questions on molecular disease mechanisms in transgenic and gene knock-out models which were subsequently widely used by the research community. This enthusiasm, however, clouded the fact that mouse models are rather imperfect in mimicking the disease process of MS (see below).

Overall, the EAE models can be classified into different types, which can be used for different specific research questions.

#### Passive T-cell transfer models

**Fig. 2:**
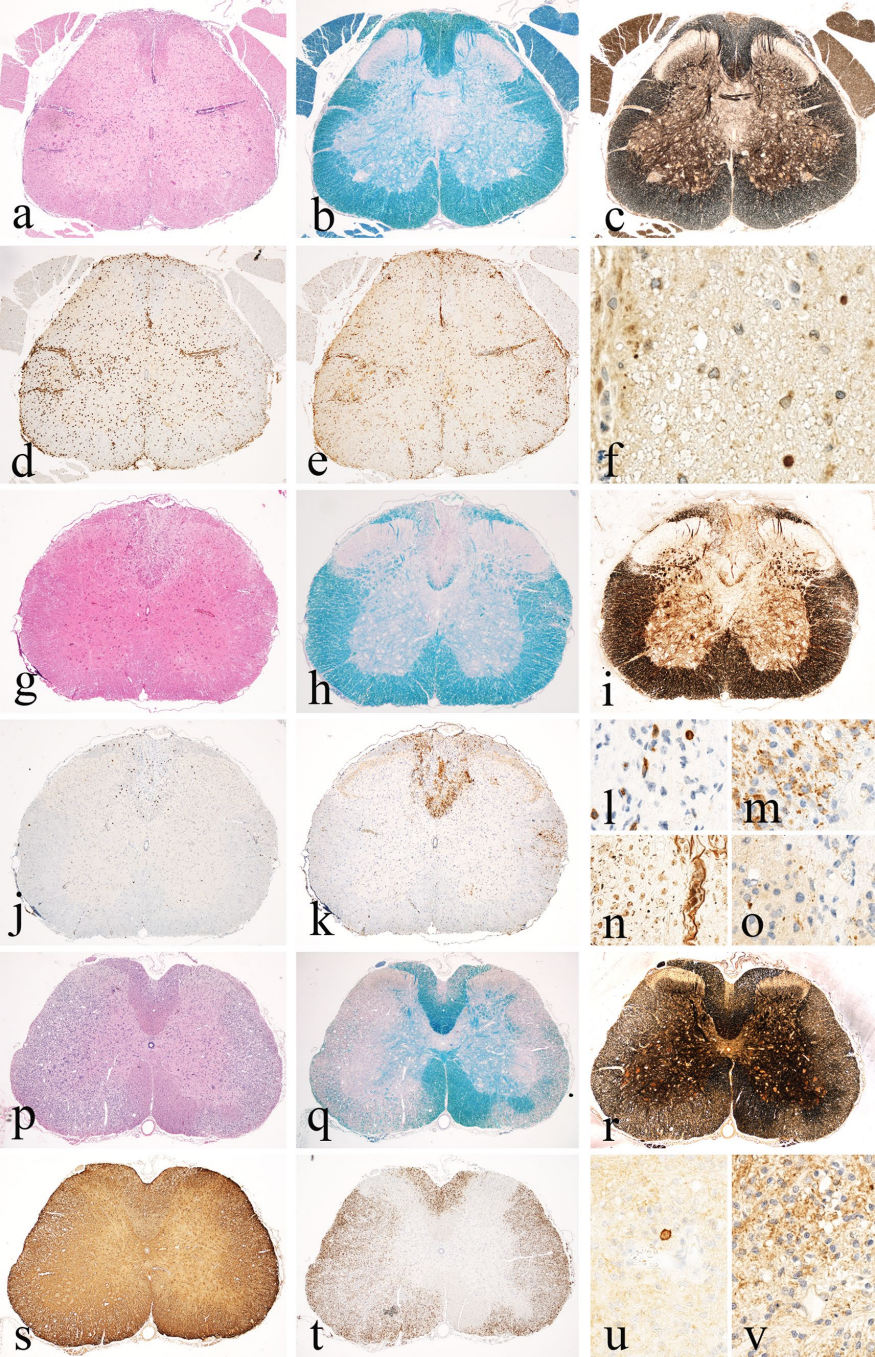
Basic patterns of pathology in different MS Models Part 1. Pure inflammatory models exemplified by passive transfer of CD4^+^ T-cells directed against myelin basic protein (MBP) in the Lewis rat. Spinal cord with massive inflammation reflected by the presence of perivenous inflammatory cuffs and diffuse infiltration of the tissue by T-cells and macrophages (**a** H&E; **d** CD3; **e** macrophage marker ED1). Sections stained for myelin (**b**, Luxol fast blue) or axons (**c** Bielschowsky silver impregnation) do not show demyelination or axonal loss, but there are some axons with accumulation of amyloid precursor protein (**f** APP), indicating a mild-to-moderate degree of (in part reversible) axonal injury. Models with chronic inflammatory axonopathy leading to focal lesions with secondary demyelination. As an example, spinal cord pathology of a NOD mouse with chronic EAE, 90 days after active sensitization with myelin oligodendrocyte glycoprotein peptide (MOG_35–55_), is shown. A confluent inflammatory demyelinated lesion is present in the dorsal column of the spinal cord (**g** H&E, **h** Luxol fast blue). There is nearly complete axonal loss within the lesion (**i**, **n** Bielschowsky silver impregnation); the lesion is infiltrated by a moderate number of T-cells (**j**, **l** CD3) and shows a broad rim of activated macrophages at the lesion edges (**k** Mac3); ongoing tissue destruction is shown by the presence of myelin protein reactive degradation products in macrophages (**m** PLP) and by the presence of numerous axons with disturbed fast axonal transport (intra-axonal accumulation of amyloid precursor protein; **o** APP). Chronic EAE in the DA rat 60 days after active immunization with full-length recombinant myelin oligodendrocyte glycoprotein as a model for extensive inflammatory demyelinating disease; profound inflammation (**p** H&E) and widespread confluent demyelination (**q** Luxol fast blue), but nearly complete axonal preservation (**r** Bielschowsky silver impregnation) and pronounced astrogliosis (**s** GFAP); the areas of active demyelination are highly infiltrated by macrophages (**t** ED1), but contain only very few T-lymphocytes (**u** CD3); and myelin sheaths and myelin degradation products in macrophages are decorated by activated complement (**v** C9neo antigen)

Pioneer work by Phillip Paterson [123] showed that lymphocytes, which were retrieved from the peripheral immune system after sensitization of animals with brain tissue, are able to induce a neuroinflammatory disease in naïve recipients. Formal proof of this observation was provided by Ben Nun, Wekerle, and Cohen [13], who were able to isolate and propagate T-cell lines and clones, specific for myelin basic protein, which triggered an encephalomyelitis in naïve recipient animals after intravenous transfer (Fig. 2). In subsequent studies, these results, originally obtained in Lewis rats, were expanded to other rat and mouse strains and to other CNS antigens, including other myelin, astrocyte, or neuronal proteins [166]. This passive transfer model is particularly useful to study the mechanisms controlling immune surveillance of the CNS, the induction of brain inflammation, and T-cell-mediated inflammatory tissue injury. These studies defined besides many other aspects the conditions, how T-cells are able to enter the CNS [46, 167], what entry routes into the brain and spinal cord they use [9, 133], how important their re-activation state is within the CNS [77, 115], and to what extent the functional polarization of T-cells determines the extent, quality, and distribution of brain inflammation [127]. A major advantage of such passive transfer models is that the T-cells are already raised and expanded, and that the outcome of brain inflammation is not influenced by the afferent arm of specific immune activation in peripheral lymphatic tissue. The major shortcoming of this model is that it is essentially restricted to inflammation, mediated by CD4^+^ T-lymphocytes. Irrespective of animals, species, or strain, and of the antigen reactivity of the T-cells used, the passive transfer of CD4^+^ T-cells induces an inflammatory disease of the CNS with some and variable axonal injury but without widespread focal primary demyelination (Fig. 2, Table 1).

As will be discussed later, such passive transfer experiments can also be performed with Class I MHC restricted CD8^+^ T-cells. Under these conditions, inflammation is associated with selective destruction of the target cells by cytotoxic T-lymphocytes, which may result in plaque-like demyelination, when the T-cells are directed against an antigen contained in oligodendrocytes [140] (Fig. 1). Similar models for auto-reactive B-cells are currently not available. They would be particularly interesting and important in the light of recent clinical trials in MS patients, showing a profound beneficial effect of peripheral B-cell depletion on the disease course [150].

#### Passive co-transfer of auto-reactive T-cells and pathogenic auto-antibodies

The intact blood brain barrier restricts diffusion of circulating auto-antibodies into the brain. In addition, auto-antibodies within the brain require immune activation of effector cells and/or the presence of complement factors forming the terminal membrane attack complex to produce tissue damage. One way to overcome this problem is to systemically inject the respective auto-antibody into animals, in which brain inflammation has been induced by passive transfer of auto-reactive encephalitogenic T-cell lines [96]. In this case, the T-cell reaction induces inflammation with activation of macrophages and microglia, and allows the antibody and complement factors to diffuse into the brain or spinal cord at sites of inflammation and blood–brain barrier damage. This paradigm has first been used to test the demyelinating potential of antibodies against myelin oligodendrocyte glycoprotein in vivo [96]. It showed that under these conditions, the inflammatory reaction is associated with widespread primary demyelination, giving rise to demyelinated plaques with close similarities to those seen in MS. This model may also shed light on the controversial discussion on the presence or absence of inflammation in active MS lesions [8] (Table 1). It showed that in the presence of high titers of circulating demyelinating antibodies, a T-cell-mediated inflammatory response in the CNS is absolutely required to trigger the demyelinating lesions, but the number of transferred T-cells, which are necessary to start the disease, can be very low. Within the respective brain lesions, only an extremely small number of T-cells are present, despite very large plaque-like active demyelination [90]. A similar pathological scenario is also seen in chronic models of MOG-induced EAE after active sensitization, when the lesions are triggered by a cooperation of encephalitogenic T-cells and demyelinating antibodies (see below).

**Table 1:** Models of inflammatory demyelinating diseases: applications and limitations in MS research

Type	Models	Characteristics	Applications	Limitations
CD4^+^ T-cell mediated inflammation	(1) Transfer of encephalitogenic T-cells [13] (2) Acute EAE induced by active sensitization with T-cell antigen or epitope [16, 111]	Highly reproducible inflammatory disease of the CNS; Inflammation by T-cells and macrophages Limited microglia activation Variable acute axonal injury Little permanent axonal loss or demyelination	Analysis of molecular mechanism involved in T-cell-mediated brain inflammation In vivo testing of anti-inflammatory treatments strategies	Only models for different CD4^+^ T-cell subsets (Th1, Th17 etc.) Relevance for inflammation in MS patients currently unclear
CD8^+^ T-cell mediated brain inflammation	(1) Passive Transfer models with: (a) true autoimmune T-cells [70] (b) artificial “neo-auto-antigen [116, 140] (2) Virus induced inflammatory demyelinating diseases [14, 37]	CNS inflammation with CD8^+^ T-cells, low macrophage recruitment, profound microglia activation Direct tissue injury induced by cytotoxic T-cells In active virus models complex interaction between CD4^+^ and CD8^+^ T-cell populations	Analysis of molecular mechanisms involved in inflammation and tissue injury induced by Class I MHC restricted T-cells Analysis of mechanisms of virus clearance from CNS and direct or bystander tissue injury	So far very difficult to handle High intra-experimental variation; The dominance of CD8^+^ T-cells in MS lesions suggests an important role, but evidences for direct CD8^+^ T-cell cytotoxicity in the pathogenesis of demyelination and neurodegeneration in MS is very limited;
Chronic CD4^+^ T-cell mediated brain inflammation and inflammatory axonopathy	Chronic relapsing or progressive mouse models after active immunization with CNS antigens; Most extensive experience in C57BL/6 mice after immunization with MOG_35–55_ [10, 60, 111]	Inflammation with focal confluent lesions, mainly in the spinal cord; Lesions with extensive axonal injury and loss and very little primary demyelination	Good model to study mechanisms of axonal injury and to test axono-(neuro-) protective treatment strategies	Value for analysis of primary de- and remyelination limited; Allows to analyze mechanisms of neurodegeneration induced by CD4^+^ T-cells and the subsequent macrophage activation; Mechanisms of neurodegeneration in MS brains are in part different; thus, validation of findings in MS is of critical importance
T-cell and antibody-mediated inflammatory demyelinating diseases	(1) Co-transfer models with encephalitogenic T-cells and demyelinating antibodies [96] (2) Active sensitization of rats, guinea pigs or primates with MOG_1–125_ [73, 89, 155, 156]	T-cell-mediated inflammation with macrophage recruitment and activation; demyelination is induced by specific demyelinating antibodies by complement or antibody-dependent cellular cytotoxicity mechanisms; Extensive primary inflammatory demyelination	The current model with the closest similarity to the pathology of multiple sclerosis (inflammation, plaques of demyelination in white and grey matter; axonal preservation, variable extent of remyelination); Similar models can be used to test the pathogenicity of other auto-antibodies, such as for instance anti-Aquaporin 4 antibodies in NMO	MS patients in general do not mount a pathogenic auto-antibody response against MOG; Patients with MOG auto-antibodies have a disease, which is different from MS; Indirect immunological and neuropathological evidence in MS argues for the presence of a humoral demyelinating (cytotoxic) factor; whether this factor is an auto-antibody or another inflammatory mediator is currently unresolved
Experimental models with inflammatory demyelination and extensive astrocyte injury or loss	(1) Virus models with additional astrocyte infection [14] (2) Models with very severe innate immunity stimulation (e.g. focal injection of LPS into the white matter [148] (3) Models with pathogenic auto-antibodies against astrocytes (e.g. NMO models) [25] (4) Toxic demyelination induced by ethidium bromide [21]	Brain inflammation with severe astrocyte injury and astrocyte loss, associated with oligodendroglia destruction, primary demyelination and axonal preservation; extensive Schwann cell remyelination	Severe astrocyte injury is present in a small subset of acute fulminant MS cases; Astrocyte injury and loss with secondary demyelination in NMO	Although astrocyte injury is present in some fulminant MS lesions, the mechanism is currently not fully defined; mechanisms identified in the respective models have to be validated regarding the relevance for MS
Viral Models of inflammatory demyelination	Theiler’s virus model [37] MHV (coronavirus) models [14]	Virus-induced inflammatory demyelinating disease, which in many aspects reflects the disease in MS	These models allow to define the mechanisms, how anti-virus and autoimmune reactions may cooperate in the induction of inflammatory demyelination	Despite extensive search so far, no MS-specific virus infection has been identified; Disease pathogenesis in these models is highly complex and proved to be difficult to dissect
Toxic Models of demyelination and remyelination	(1) Cuprizone Models [132] (2) Lysolecitin Model [74] (3) Ethidium bromide Model [21]	Highly reproducible time course of demyelination and remyelination; well-defined pathophysiological mechanisms of demyelination	Very good models to study basic biology of demyelination and remyelination	Very efficient spontaneous remyelination after cessation of the toxic injury; permanent remyelination failure, as seen in many MS lesions, is only seen in models with prolonged cuprizone intoxication

More recently, this model has also been used to define the pathogenetic potential of other antibodies, such as those directed against neurofascin or against aquaporin 4 [25, 109]. The latter is now an important experimental model for neuromyelitis optica (NMO), which allows to define the pathogenetic role not only of patient-derived anti-aquaporin 4 auto-antibodies, but also the role of aquaporin 4 reactive T-lymphocytes [129, 169]. A limitation of this model for NMO is that it has been so far not possible to raise a stable pathogenetic “true-autoimmune” aquaporin 4 antibody response in rats or mice by active sensitization. In one of the latest attempts, reported by Kurosawa et al. [85], pathogenic murine aquaporin 4-specific antibodies were produced by immunization of aquaporin 4 deficient mice. Thus, experiments are most frequently performed with human antibodies, which induce an anti-human immunoglobulin G response, which may interfere with pathogenetic mechanisms in chronic disease conditions.

#### Active sensitization models

These models require active immunization with a CNS antigen together with a strong adjuvant (such as for instance complete Freund’s adjuvant, CFA [11, 75]). In mice, this alone is generally not sufficient and additional treatment of the animals with pertussis toxin is necessary [16]. The exact mechanism, how pertussis toxin augments the sensitization process, is still largely unknown. Suggested potential mechanisms are the inhibition of peripheral T-cell anergy induction [76], a suppression of the function of regulatory T-cells [29], an effect on innate immunity [41], or direct effects on the blood brain barrier [99]. The most frequently used active sensitization model is induced in mice by sensitization with the MOG_35–55_ peptide in CFA [111] (Fig. 1). This model is easy to induce and results in an acute or chronic inflammatory disease in the spinal cord, dependent upon the immunization procedure and the genetic background of the animals. Although this model is most popular for the analysis of molecular disease mechanisms in transgenic mouse strains, it has major limitations as a model for MS. It is a model for an acute or chronic inflammatory encephalopathy with primary axonal injury mediated by auto-reactive MHC Class II restricted CD4^+^ T-cells [78; 151; 118). Larger lesions are mainly due to massive axonal degeneration with secondary demyelination, while primary demyelination is sparse or absent, a pathological picture that is quite different from that seen in MS (Fig. 2). Another shortcoming of this model is that its pathology is largely confined to the spinal cord, with low affection of the brain stem and the cerebellum and very little inflammation or tissue damage in the forebrain.

A disease, which more closely resembles MS, is induced by active sensitization of rats [156], guinea pigs [89], or primates [73] with the recombinant extracellular domain (amino acids 1–125) of myelin oligodendrocyte glycoprotein (MOG), with myelin or with brain tissue in CFA. The pathogenetic principle in these models is that they induce a CD4^+^ T-cell-mediated encephalitogenic response together with a demyelinating auto-antibody response against MOG [96]. The pathological hallmark in these models is the appearance of large confluent plaques of primary demyelination with partial axonal sparing, comparable to the lesions seen in MS patients (Figs. 1, 2) [156]. The extent of demyelination and the topographical distribution of the demyelinated lesions depend upon the genetic background of the animals [165]. Thus, in certain rat strains and in primates also, very large (subpial) cortical demyelinated lesions are seen, which are related to a chronic inflammatory process in the meninges [131, 155] (Fig. 1). Overall, these models in many respects resemble the pathological changes in MS, although it has to be considered that anti-MOG antibodies are, in general, not present in the serum of MS patients. When they are found in patients, they are mostly associated with clinical phenotypes, which are distinct from MS, resembling ADEM, aquaporin 4 negative NMO, or an atypical inflammatory demyelinating disease, which does not fulfill the diagnostic criteria for MS [79, 147]. Thus, MOG-induced disease in experimental animals may be a perfect model for a MOG autoimmune syndrome in humans, which may be distinct from MS. In MS, demyelinating and neurotoxic activity have been described in sera and cerebrospinal fluid. Whether this is due to auto-antibodies against so far undefined CNS antigens [23] or to inflammatory mediators other than immunoglobulin [98] is currently unresolved (Table 1).

#### Spontaneous models of EAE

Diseases, which are induced by passive transfer of T-cells or by active immunization, offer only limited possibilities to identify immunological mechanisms, which are responsible to initiate the disease process or which regulate relapses. To overcome these problems, new models have been created, in which animals develop a spontaneous inflammatory demyelinating disease. This can be done by transgenically expressing a T-cell receptor of encephalitogenic T-cells, which recognizes a brain antigen, such as MOG [17]. These animals are born normal and when kept under the conventional breeding and housing conditions develop a spontaneous inflammatory or inflammatory demyelinating disease several months after birth (Fig. 1), in a frequency of up to 80 %, depending on mouse strain and gender [130]. In addition, such animals can be crossed with B-cell receptor transgenic mice, which mount an auto-reactive (demyelinating) antibody response [18, 83]. However, despite the presence of potentially demyelinating antibodies in these double transgenic mice, primary demyelination in the CNS lesions is sparse in contrast to those in rats or primates with a demyelinating anti-MOG antibody response and no complement activation is seen in the lesions [83]. As in other mouse models, the pathology of the lesions shows preferential axonal loss with secondary demyelination. Thus, mouse complement activation appears to be less efficient in comparison to that of rats, guinea pigs, or primates. This view is also supported by the observation that the induction of lesions in the mouse after focal injections of (human) aquaporin 4 or MOG antibodies requires the simultaneous presence of human complement [138, 139].

Spontaneous EAE models were found particularly useful for research on the role of gut microbiota in the induction of brain inflammation [15]. When such animals are kept under germ free condition, no disease develops. However, disease is triggered when their gut is colonized with the normal gut flora. Such models can be used to identify the bacterial components of the gut flora, which trigger and expand an encephalitogenic T-cell response. Another interesting observation in such a model was, that in SJL mice, but not in C57BL/6 mice, the presence of the transgenic encephalitogenic T-cells may trigger an endogenous demyelinating anti-MOG antibody response [130]. These results help to explain the mechanisms of antigen/epitope spreading in the induction of chronic brain inflammation, which has been shown in several other EAE models before. Overall, however, the same limitation, as discussed before, applies also for these models. They describe CD4^+^ T-cell-mediated inflammatory processes of the CNS. As in other mouse models, demyelination is mainly following axonal pathology with a little primary demyelination. To what extent findings obtained in these models are relevant for MS has to be shown in studies involving patients.

#### Humanized EAE models

Species-related differences in the molecular function of proteins are a problem, which require transgenic expression of the respective molecules in experimental animals in vivo. Since myelin proteins are highly conserved between different species, this problem essentially concerns antigen presentation. As an example, T-cells only recognize their respective antigen, when it is processed in antigen presenting cells and presented in the context of major histocompatibility complex (MHC) molecules, which are highly polymorphic and different between vertebrate strains or species. Thus, the determination of the encephalitogenic potential of auto-reactive T-cells, identified in patients, has to be validated in animals, which express the respective human MHC proteins. This can be achieved in humanized mouse models [12, 42]. Respective studies of EAE, induced in human MHC Class I or II transgenic animals, have contributed to define antigenic peptides, which may be encephalitogenic in humans. However, encephalitogenicity is not only regulated by antigen presentation, but also involves antigen processing and the involvement of adhesions molecules and chemokines, necessary for T-cell migration into the CNS. Thus, an ideal experimental design would require multiple transgenic manipulations, in which all the molecules involved in this process are humanized. Similar rules also apply for the validation of any other molecular pathway involved in the pathogenesis of a disease, such as MS. It is obvious that such an approach is highly complicated and technically challenging, and so far, its use in experimental pathogenesis research is very limited.

#### Models of focal inflammatory demyelinating lesions in the CNS

All the models described above show inflammatory demyelinating lesions, which are dominantly located in the spinal cord. The location of the lesions is randomly distributed with a certain predilection for the lumbar/sacral spinal cord, but for analyzing molecular events in vivo by dual photon laser microscopy or functional changes by electrophysiology, the precise and reproducible localization of the lesion within a specific area of the CNS is required. It has already been shown in the earliest studies on EAE that inflammatory lesions are precipitated into areas of concomitant brain injury, such as direct trauma, thermal injury lesions, or cyanide-induced demyelinating lesions [92, 93]. This has recently been expanded by the observation that inflammation is also precipitated into demyelinated lesions induced by cuprizone [143]. Interestingly, when EAE pathology is precipitated into forebrain lesions, this is associated with less severe spinal cord or brain stem lesions [92]. Thus, the shift of EAE lesions from a site relevant for clinical deficit into a clinically silent area, which may happen after focal injection of a therapeutic agent, such as, for instance, stem cells, into the forebrain may erroneously mimic therapeutic efficacy.

A similar approach has been used by focal stereotactic injection of a small amount of pro-inflammatory cytokines in animals, which are pre-sensitized with MOG_1–125_. The latter induces an encephalitogenic T-cell response and a demyelinating anti-MOG antibody response in the peripheral immune system of the sensitized animals (Fig. 1). Focal injection of cytokines precipitates inflammatory demyelination at a defined site of the CNS, located around the injection site. As an example, this strategy was used to define, in detail, the cellular events occurring in cortical EAE lesions [54, 112].

Focal CNS injections can also be used for testing the pathogenic potential of inflammatory mediators, which do not sufficiently pass the intact blood brain barrier in normal animals. This has, for instance, been used for testing the pathogenic potential of demyelinating or aquaporin 4 reactive auto-antibodies in vivo and to analyze the effector mechanisms, such as complement activation or antibody-dependent cellular cytotoxicity, in the process of antibody-mediated tissue injury [138, 139, 162].

Using a comparable approach, mechanisms of innate immunity in the pathogenesis of demyelination have been elucidated. Focal injection of lipopolysaccharide into the spinal cord induces a transient inflammatory reaction during the first days after application, which is followed by the formation of focal inflammatory demyelinating lesions after a delay of several days [43, 106]. The latter is associated with profound microglia and macrophage activation, profound alterations of astrocyte function [148], and a mechanism of tissue injury, which involves oxygen radical-mediated cell injury and hypoxia-like cellular damage, thus, reproducing some key features of lesion pathology seen in a subset of multiple sclerosis patients and lesions [38].

#### EAE models for primary or secondary progressive MS

After active immunization, EAE animals develop either an acute monophasic or a chronic disease, which may last for several months. In the Biozzi ABH mouse, the chronic course may present with relapses or remission [7] or a disease, which has been described as chronic progressive [3, 60]. However, in these models, the chronic disease is not clinically progressive but reflects stable disease on a moderate-to-high level of neurological disability. In an attempt to reproduce the profound axonal and grey matter injury seen in progressive MS, Biozzi mice were sensitized with neurofilament-L [68, 69]. This procedure resulted in an acute or subacute neuroinflammatory disease with severe axonal pathology also targeting the grey matter, apparently mediated by CD4^+^ T-cells directed against neurofilament. However, also this model did not reproduce the slow progressive disease characteristic for the progressive stage of MS. More recently, it has been shown that a CD4 T-cell-driven autoimmune disease is also induced due to molecular mimicry between myelin oligodendrocyte glycoprotein_35–55_ and neurofilament-L [84]. Whether, in these models, neurodegeneration is due to direct interaction between auto-reactive T-cells and neurons or by activated microglia is currently unresolved.

Another study analyzed the effect of age in the induction of autoimmune encephalomyelitis in Biozzi mice [125]. This study reports the induction of a primary progressive like disease course, when animals were sensitized with spinal cord homogenate in complete Freund’s adjuvant, although details on the clinical course are not provided in this study. In contrast to the situation in primary progressive MS in humans, this disease was characterized by very prominent T-cell infiltration in the lesions, which exceeded that seen in comparable models of relapsing or secondary progressive disease.

Profound microglia activation is associated with progression of demyelination and neurodegeneration in the brain of MS patients. One attempt to create a model for progressive MS was, thus, to induce EAE in mice with a genetic background of high innate immunity activation. This was achieved using the non-obese diabetic (NOD) mouse strain [10] (Figs. 1, 2). Such animals develop chronic, in part progressive EAE, over a time period of 70 days with an incidence of 90 % [94, 95]. Although these mice have large lesions in the spinal cord, the pathological alterations are not essentially different from those seen in other mouse EAE models (inflammation, dominant axonal injury with secondary demyelination, and little primary demyelination). As in MS, oxidative injury appears to play a major role in chronic tissue injury, shown by the neuroprotective action of anti-oxidant treatment [10].

One hypothesis, explaining neurodegeneration in progressive MS is that inflammation starts a cascade of events in the brain, which leads to ongoing neurodegeneration that becomes independent from the inflammatory process. This question was addressed in the chronic EAE model in Biozzi/ABH mice described above. In this model, inflammation was blocked by tolerization with a CD4 T-cell-depleting monoclonal antibody at different stages of the chronic disease [60]. This study shows effective reduction in the inflammatory response, absence of active demyelination, and increased remyelination in the tolerized animals. Despite these findings, evidence was provided for ongoing and progressive axonal and neuronal degeneration up to 50 days after tolerization [60]. Ongoing axonal and neuronal degeneration was not reflected by a chronic progressive worsening of neurological disease. In this context, one has to consider that after the transection of axons within a focal lesion, Wallerian degeneration and retrograde or anterograde neuronal degeneration take place and this is a very slow process. Thus, the detection of axonal pathology and progressive loss of axons and neurons may merely reflect the late consequences of lesions, which developed during the active phase of the disease, but do not model progressive disease in MS.

Cortical (subpial) demyelination is a key feature of the pathology of progressive MS. It is already seen in the early disease stages [101] but massively increases, when patients develop progressive disease [86] (Fig. 1). Cortical demyelination is associated with neuronal degeneration [102] and this appears to be a major driving force for secondary Wallerian degeneration in the white matter. In fact, diffuse pathology of the white matter in MS patients correlates better with the extent of cortical lesions than with the size, destructiveness, and location of white matter plaques [86]. Thus, cortical pathology appears to be a very important driving force for chronic progressive neurodegeneration in the MS brain. Active cortical demyelination occurs at sites of meningeal inflammation. In patients with severe and aggressive disease, the inflammatory aggregates in the meninges structurally resemble tertiary lymph follicles [102]. The question, thus, arises to what extent meningeal inflammation and subsequent subpial cortical demyelination can be modeled in EAE animals.

In most chronic EAE animals, meningeal inflammation is sparse and cortical demyelination is absent. There are, however, some exceptions. In specific MOG immunized mouse strains, extensive meningeal inflammation with formation of tertiary lymph follicle-like structures is seen. The formation of these follicles is augmented by the expression of podoplanin in Th_17_ T-cells and in meningeal cells, a molecule which is crucial for the development of secondary lymphoid follicles [126]. However, meningeal inflammation in this mouse model was not followed by MS-like subpial demyelination.

Extensive subpial demyelination, associated with meningeal inflammation, has been found in selected EAE models in rats and primates after active sensitization with MOG_1–125_ [131, 155] (Fig. 1). More detailed immunopathological studies of such lesions suggest that they are triggered by inflammatory mediators in cooperation with demyelinating antibodies, which, both, are produced in the meningeal inflammatory infiltrates, diffuse into the cortex, and induce demyelination at distant sites in association with microglia activation. A very similar scenario has been suggested to explain the pathogenesis of subpial cortical lesions in MS patients. Active cortical lesions are associated with meningeal inflammation, which, in its most severe form, may reflect inflammatory cell aggregates with features of tertiary lymph follicles. Whether cortical lesions in MS patients are driven by demyelinating auto-antibodies, which have been detected in the cerebrospinal fluid of MS patients [23], or by other so far unidentified inflammatory mediators [98] is currently not clear.

Recently, chronic microglia activation, excessive oxidative stress, mitochondrial injury, and amplification of oxidative stress by iron liberation from degenerating oligodendrocytes have been suggested as a major driving force of demyelination and neurodegeneration in MS, and this mechanism seems to be particularly important in the progressive stage of MS [103]. Similar tissue alterations are, in part, also present in EAE models, but there are major differences in their degree in comparison to MS. Expression of molecules involved in oxygen radical production is prominent in macrophages within the EAE lesions, but in contrast to the human CNS, they are sparse or absent in microglia at the lesion edge of the normal appearing white or grey matter [144]. Axonal degeneration in acute mouse EAE models involves oxidative stress and mitochondrial injury [118], but the widespread mitochondrial injury with deletion of mitochondrially encoded genes, which is a characteristic finding in MS [28, 104], has so far not been seen in EAE. Finally, iron accumulation in myelin and oligodendrocytes occurs in an age-dependent manner in the human brain and iron is liberated into the extracellular space in actively demyelinating MS lesions [59]. In rodents, a limited degree of iron accumulation is seen at the end of the animal’s life span in the basal ganglia, but not in the rest of the brain or the spinal cord, typically targeted by the inflammatory process in EAE, and it is completely absent in young adult animals preferentially used in EAE experiments [144].

For all these reasons, progressive MS is reflected only to a very limited degree in currently available EAE models.

#### EAE models driven by MHC Class I restricted CD8^+^ T-cells

The abundance of CD8^+^ T-cells in MS lesions suggests that these cells may play an important role in the pathogenesis of the disease. So far, however, it proved to be very difficult to induce a pathogenic CD8^+^ T-cell autoimmune response by active immunization. One observation, which suggests that this may be possible, came from a study, which analyzed, in detail, the immunology and immunopathology of EAE in C57BL/6 mice, induced by sensitization with MOG_35–55_ [158]. In this study, a prominent MOG-specific CD8^+^ T-cell response was observed and CD8^+^ T-cells, although not in a completely pure form, modified brain inflammation after passive transfer. A contribution of MOG-specific CD8^+^ T-cells to brain inflammation has also been reported in T-cell receptor transgenic animals [2] and in a humanized mouse model [107], (Table 1).

Another approach isolated and propagated auto-reactive CD8^+^ T-cells from mice, actively sensitized with myelin basic protein, and transferred them into naïve recipients. This induced clinical disease and brain inflammation in the recipient animals [70]. In other studies, foreign antigens (ovalbumin or influenza hemagglutinin) were transgenically expressed in different cells of the CNS and disease was induced by the passive transfer of T-cells from transgenic animals, which express a T-cell receptor directed against the respective pathogenic epitope of ovalbumin or hemagglutinin [27, 116, 140] (Fig. 1). Taken together, these studies show that auto-reactive Class I MHC restricted T-cells can induce brain inflammation without the help of other T-cell populations in the immune repertoire. The studies have further shown that these CD8^+^ T-cells are cytotoxic and destroy the cells within the CNS, which contain the cognate antigen. In case of CD8^+^ T-cells recognizing an antigen within oligodendrocytes, this leads to focal plaques of primary demyelination and oligodendrocyte loss, but other models, targeting astrocytes [27] or neurons, are also available [143].

However, transfer of CD8^+^ T-cells in general requires much higher cell numbers to induce brain inflammation and tissue injury compared with CD4^+^ T-cells. This may in part be explained by the observation that oligodendrocyte-specific CD8 cells can enter the normal brain and be eliminated by apoptosis in the course of their interaction with normal oligodendrocytes. However, when they enter the inflamed brain, they are cytotoxic [117]. Using humanized transgenic mouse models, it has been shown that CD8^+^ T-lymphocytes carrying a human T-cell receptor against proteolipid protein, which has been derived from T-lymphocytes of an MS patient, also can induce brain inflammation in vivo [51]. Overall, however, data on the pathogenicity of auto-reactive CD8^+^ T-cells are limited in comparison to those available for CD4^+^ cells.

A major unresolved question of experimental MS research is that currently, no models of B-lymphocyte-associated brain inflammation are available. It is not clear, whether B-cells alone may initiate and drive an acute or chronic inflammatory reaction in the CNS, whether they are just secondarily recruited into T-cell-mediated inflammatory foci and in what way they contribute to the induction and propagation of tissue injury, independent from antibodies.

### Models of virus-induced inflammatory demyelination

EAE is an autoimmune disease of the CNS, which is induced by active immunization with brain antigens together with potent immune-stimulating adjuvants, by passive transfer of primed T-cells or by transgenic expression of an autoantigen recognizing T-cell receptor. None of these scenarios give clues on how the disease may be triggered in MS patients. It was, thus, interesting to see that certain virus infections in animals may give rise to an inflammatory demyelinating disease with similarities to MS [37]. So far, however, no MS-specific virus infection has been found, although some indirect evidence suggests that infection with Epstein Barr Virus or activation of endogenous retroviruses may play a role in its pathogenesis [4, 120].

Despite the current lack of evidence for an MS-specific virus infection, the study of virus induced experimental models of inflammatory demyelination may provide insights into the basic mechanisms, how such a pathological condition in the brain and spinal cord could be induced or propagated, and such studies may provide additional information on mechanisms of MS-related inflammation, demyelination and neurodegeneration [37]. A major drawback for virus models, however, is their very complex pathogenesis, involving direct virus induced effects, anti-viral immunity, and additional autoimmune mechanisms [37], and the effects of these different pathogenetic components are difficult to dissect. The two most widely used viral MS models are chronic encephalomyelitis induced by the Theiler’s virus or by the MHV corona virus.

#### Chronic demyelinating encephalomyelitis induced by the Theiler’s virus (TMEV)

The model is induced by the direct intracerebral infection of the animals with the virus. This leads to an acute encephalomyelitis in most infected animals, and disease course and mortality depend upon the virulence of the virus strain used for infection and the genetic ability of the host animals to mount a specific anti-viral T-cell response. Using specific virus strains (BeAn strain or Daniel’s strain) in mice with specific MHC haplotypes (H-2^q,r,s,v,f,p^), the acute encephalitic phase is followed by a chronic demyelinating disease, which mainly affects the spinal cord [37]. Virus antigen is cleared from the brain, but persists in the spinal cord in oligodendrocytes [136] and macrophages [97]. The spinal cord lesions are characterized by chronic inflammation, the formation of confluent plaques of primary demyelination, a variable extent of axonal injury, and remyelination, the latter dependent upon the genetic background of the mouse strain [19, 137]. Inflammatory infiltrates consist of a mixture of CD4^+^ and CD8^+^ T-cells, B-cells, and plasma cells [119, 122]. Active demyelination occurs at sites of microglia activation and macrophage infiltration. Thus, the lesions share essential features with those present in MS (Table 1).

The TMEV model has extensively been used to study the mechanisms of viral clearance from the CNS. It has been shown that in the early stage, the immune response is mainly directed against the virus [114], but that at later stages, additional autoimmune reactions are induced, most likely through the mechanisms of antigen/epitope spreading [121]. The model also made it possible to define the role of different lymphocyte populations in the pathogenic process. While CD8^+^ T-cells were instrumental for virus clearance together with a virus-specific antibody response, CD4^+^ T-cells were involved in inflammatory demyelination [114]. In contrast, depletion of CD8^+^ T-cells reduced chronic disease in parallel with chronic axonal injury and neurodegeneration [67, 134]. A major difference to what is seen in MS is that a B-cell depleting therapy augments the early inflammatory as well as the late inflammatory demyelinating disease, and this was associated with an increase in viral load within the CNS [55]. The complexity of the disease process in the TMEV models is also reflected by the outcome of treatment studies with induced regulatory T-cells [108]. Regulatory T-cells increased disease in the early stage of the disease by suppressing the anti-viral response, but showed a beneficial effect in the late inflammatory demyelinating stage.

#### Chronic inflammatory demyelinating disease induced by the Mouse Hepatitis (corona) Virus (MHV)

The isolation of MHV from the brain of a paralyzed mouse, presenting with disseminated encephalomyelitis and prominent demyelination [5], suggested that inflammatory demyelinating diseases may be triggered by viral infection. In the subsequent years, MHV-induced demyelinating encephalomyelitis was developed as a major experimental animal model of MS. MHV is a large corona virus, which may induce hepatic, enteric, respiratory, or neurological disease, depending upon the virus strain. Neurological disease occurs after intracranial or nasal infection with a neurovirulent strain [14]. It develops in two phases: the first phase starts within days after infection and results in a virus-induced panencephalitis. When the animals recover from this first phase, a second phase may appear about 4 weeks later, characterized by neuroparalytic disease with inflammatory demyelinating lesions. Virus is largely eliminated from the brain at the end of the first phase, but viral RNA persists within the late lesions throughout the entire disease. The virology of JHM-induced brain disease has recently been summarized in an excellent review [14]. For this reason, we will only discuss some aspects related to the chronic inflammatory demyelinating disease within this chapter.

**Fig. 3:**
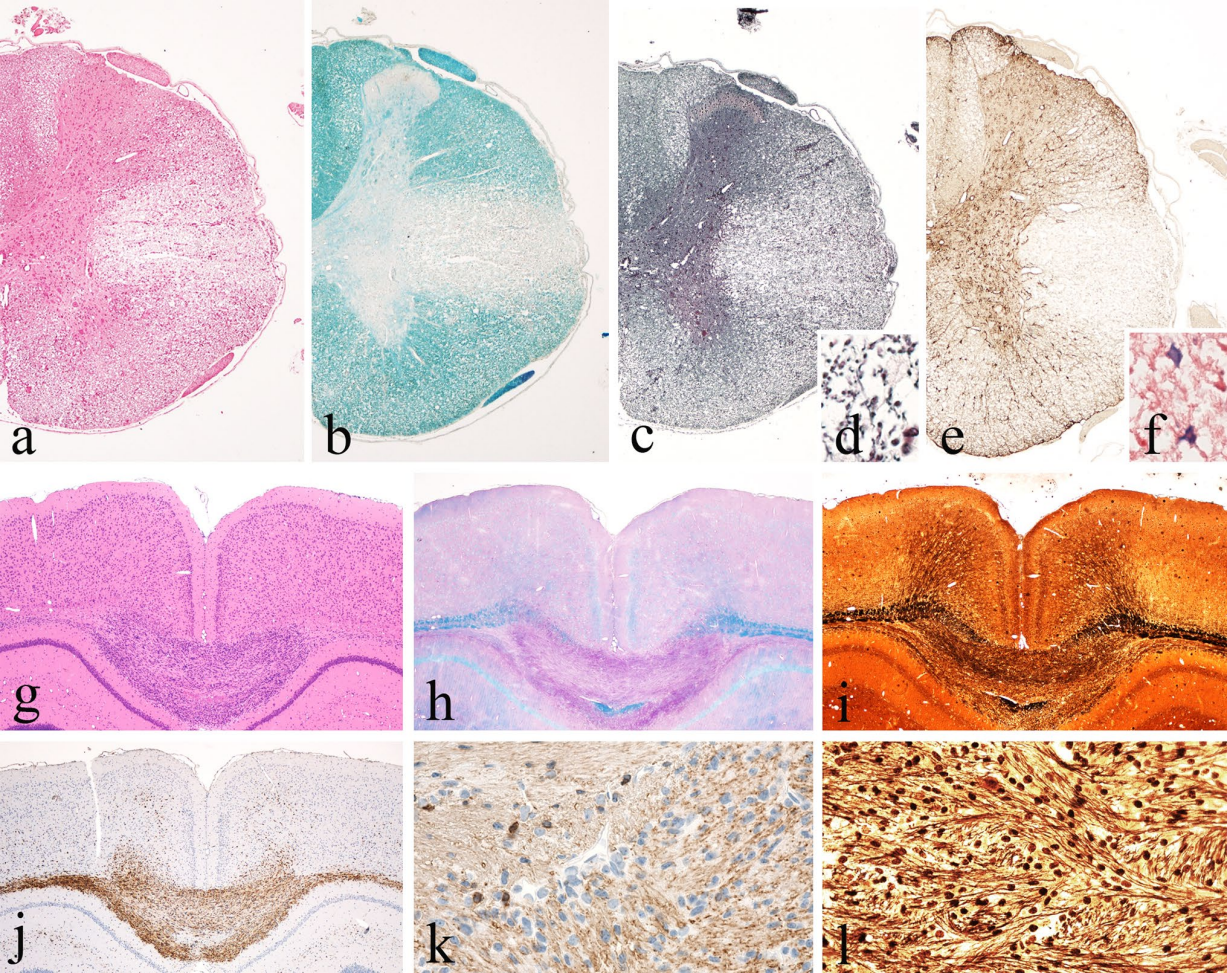
Basic patterns of pathology in different MS models Part 2. MHV-induced spinal cord pathology as an example for inflammatory demyelinating lesions with extensive oligodendrocyte and astrocyte loss; large confluent demyelinated lesions in the lateral column of the spinal cord with inflammation and tissue edema (**a** H&E), complete demyelination (**b** Luxol fast blue), and nearly complete axonal preservation (**c**, **d** Bodian silver impregnation), but nearly complete loss of astrocytes (**e** GFAP); some of the astrocytes at the lesion edge contain virus antigen (**f**). The cuprizone model as an example for toxic demyelination; large hyper-cellular demyelinating lesion in the corpus callosum after 6 weeks of cuprizone exposure (**g** H&E). The lesion shows complete demyelination (**h** Luxol fast blue) and only mild or moderate axonal loss (**i**, **l** Bielschowsky silver impregnation); the site of active demyelination is highly infiltrated by macrophages and activated microglia (**j** Mac3); oligodendrocytes are lost within the areas of active demyelination and only preserved in the areas with intact myelin (**k** CNPase)

The pathology of the disease is characterized by chronic inflammation and the formation of confluent plaques of primary demyelination with variable extent of acute axonal injury and axonal loss (Fig. 3). Virus antigen is seen mainly in the acute phase of the disease and is present in multiple different cell types, including macrophages, microglia, astrocytes, oligodendrocytes, and neurons. When the animals recover from the acute phase, virus in the brain is largely cleared, although viral RNA persists in the tissue throughout the chronic demyelinating phase. However, viral protein expression is sparse or undetectable [153]. Inflammatory infiltrates consist of T-lymphocytes and activated macrophages/microglia [154, 168]. Intrathecal production of immunoglobulins also suggests a major contribution of B-cells and plasma cells to the inflammatory process [39]. Various different immune mechanisms contribute to virus clearance from the CNS, including innate immune mechanisms, CD8^+^ and CD4^+^ T-cells, and anti-viral antibodies. Originally, it was suggested that demyelination is a result of lytic oligodendrocyte infection [88], but with application of immune-deficient animal models, it became clear that immune-mediated mechanisms may be more important [66]. They include antigen-independent (bystander) destruction of oligodendrocytes by CD8^+^ T-cells [34, 36] and gamma-delta T-cells [35] or recognition of virus antigen on oligodendrocytes by specific anti-viral antibodies [80] (Table 1). The mechanisms of tissue injury may, however, in part depend upon the genetic background of the animal and the virus strain of MHV. From all these data, there is strong support for the view that the immune mechanisms, which are involved in virus clearance and the induction of inflammatory demyelination, are different. This view is also supported by the finding that treatment of MHV-infected animals with the immunomodulatory drug fingolimod (FTY720) increases disease severity and viral load in the CNS, but reduces the severity of the demyelinating process [22]. Whether the immune-mediated damage is due to a bystander effect of inflammation or to virus-induced autoimmunity is currently not resolved. However, it has been shown that MHV infection may trigger an encephalitogenic T-cell-mediated autoimmune response against myelin basic protein [164] or proteolipid protein [33]. An interesting difference between MHV models and EAE is that the chronic virus-induced inflammatory demyelination is associated with a massive microglia activation, which is seen in the entire brain and spinal cord, and is characterized by expression of oxygen radical producing enzymes in microglia even at the lesion edge and in the normal appearing white matter, and by extensive oxidative injury in active lesions [144]. In addition, profound astrocyte degeneration may be present, possibly due to virus infection of these cells. Astrocyte dysfunction may contribute to the pathogenesis of oligodendrocyte loss and demyelination. As in toxic models of demyelination with profound astrocyte degeneration, such lesions are preferentially repaired by Schwann cell remyelination.

Until recently, JHM infection has been a common threat in laboratory animal (rodent) colonies [65]. Although this most commonly took place with enteric virus strains, which do not affect the CNS, some caution is warranted. In a study on EAE, induced by passive transfer of auto-reactive T-cells, very unusual large and destructive lesions were noted in the spinal cord. This was present in animals with MHV antibodies in the circulation and further virological analysis revealed coronavirus replication in the lesions [32].

One of the major advantages of virus-induced models of inflammatory demyelinating diseases is that they are caused by the infectious process and not by an active sensitization with brain tissue. This may be a more natural process providing information on the etiology of the disease process in humans. However, despite extensive investigations of these models, key aspects have so far remained unresolved. It is likely, but not finally proven, that the chronic inflammatory process in these models is maintained by the persistence of low amounts of virus in the brain, but that the further tissue damage involves adaptive and innate immune mechanisms, different from those responsible for infection control.

### Models of toxic demyelination

Primary demyelination is a pathological hallmark, distinguishing MS from other inflammatory diseases of the central nervous system. To identify new therapeutic strategies, targeting de- and remyelination requires in depth knowledge of the mechanisms involved in these processes [50]. Such information can best be obtained in models of toxic demyelination, which are not complicated by changes in the CNS due to inflammatory processes driven by adaptive immunity. The most frequently used model of toxic demyelination is induced by systemic exposure of the animals to cuprizone [56, 132]. Other models include the focal injection of lysolecithin [58, 74] or of ethidium bromide into specific white matter tracts [21]. Research in these models has provided seminal insights into metabolic processes involved in myelin destruction and repair (for review, see: [49, 50, 132], and was important for in vivo imaging of demyelinated lesions by magnetic resonance imaging [128], for determining the neuroprotective effect of remyelination [49], and for testing therapies aimed to promote remyelination (Table 1).

#### Cuprizone induced de- and remyelination

The basic features of the cuprizone model, including metabolic and inflammatory mechanisms of de- and remyelination, have recently been summarized in detailed and comprehensive review articles [56, 132], and therefore, only some key characteristics related to the value of this model for MS research will be covered here. Cuprizone is a copper chelating drug, which triggers apoptosis in oligodendrocytes and induces demyelination through mechanisms of oxidative injury. The processes of de- and remyelination are further amplified or modified by inflammatory mechanisms, involving microglia and astrocytes [56]. Most models apply cuprizone in C57BL/6 mice for 4 weeks, which induces demyelination followed by rapid and extensive remyelination [63]. However, demyelinating lesions can be induced in many different mouse strains and also in other animal species, such as, for instance, rats, guinea pigs or hamsters [132]. The predictable location in the corpus callosum (Fig. 3) and time course of the lesions in this model provide an excellent background to define the molecular mechanisms promoting de- and remyelination. However, the rapid and extensive spontaneous remyelination after short-term cuprizone exposure imposes limitations for the use as an experimental MS model. Thus, when using this model in the development of new remyelination-promoting therapies, it has to be kept in mind that the respective studies show an acceleration of the remyelinating process, but do not correct for a permanent remyelination failure characteristic of MS lesions particularly in the progressive stage of the disease. However, even when the cuprizone lesions become fully remyelinated, progressive neurodegeneration may ensue. This is reflected by long-term and progressive decline in motor performance of the animals, associated with ongoing axonal injury in (re)myelinated fibers [105].

To overcome the problem of rapid remyelination, a second model has been developed by exposure of animals to cuprizone for 12 weeks. In this model, chronic demyelinated lesions are induced with little remyelination [110]. Impaired remyelination seems to be due to different factors, such as the depletion of the oligodendrocyte progenitor cell population, changes in the focal environment of cytokines involved in proliferation, or differentiation of progenitor cells or factors which inhibit their migration into the lesions [132]. Similar factors have been proposed to explain in part remyelination failure in MS and have also been implicated in age-related decline of the remyelination capacity [40, 149].

#### Models of focal toxin-induced demyelination

Lysolecithin, injected into white matter tracts of the CNS, induces focal plaques of demyelination due to a direct (detergent) action of the toxin, which damages the lipid membrane-rich myelin sheath [74]. It is a highly reproducible model of primary demyelination, which has the advantage of triggering a focal lesion at a defined location within the CNS. As in other toxic models of demyelination, the phase of myelin destruction is rapidly followed by remyelination, although the speed and degree of remyelination are age-dependent [30, 48]. Age-related insufficiency of remyelination appears to be due to epigenetic control of oligodendrocyte differentiation inhibitors [149], to impairment of the recruitment of different oligodendrocyte progenitor subsets [31], and to insufficiency of macrophages to clear the remyelination-inhibiting myelin debris [82]. These age-related effects on remyelination capacity appear to be highly relevant for remyelination failure in the progressive stage of MS.

Another focal model of demyelination used the focal injection of ethidium bromide into white matter tracts [21]. This leads to degeneration not only of oligodendrocytes but also of astrocytes. With this model, it was shown that oligodendrocyte remyelination requires the presence of astrocytes. When absent, the lesions are repaired by Schwann cells. This may explain the extensive Schwann cell remyelination seen in some patients with neuromyelitis optica [71].

There is no doubt that toxic models provided the major clues for the understanding of molecular mechanisms of de- and remyelination. They showed that remyelination not only improves the function of axons, but also that it is neuroprotective. They also showed that the mechanisms of remyelination are highly complex and require the presence of progenitor cells, which are capable to perform the task, and a finely tuned expression of stimulatory and inhibitory cytokines or growth factors. To determine the extent, to which these factors or mechanisms are relevant for MS requires analysis of respective lesions in the patients and the careful validation of the therapeutic potential of the factors in MS in clinical trials.

## Conclusions

Although no single experimental model reproduces all aspects of multiple sclerosis in a single animal disease, a very large spectrum of different models is currently available, which covers individual aspects and mechanisms relevant for disease pathogenesis. The selection of the right model for MS research, thus, largely depends upon the specific question, to be addressed. Regarding testing of new therapeutic strategies, these models may provide proof of principle that the respective approach provides positive effects in vivo, but many treatments tested positively in these experimental models failed in human clinical trials. However, such studies are indispensable to recognize as far as possible potential harmful side effects of new drugs in the inflamed or damaged CNS.

There are, however, key aspects of multiple sclerosis pathology and pathogenesis, including the role of CD8^+^ T-cells and B-cells, and the mechanisms of demyelination and tissue damage in the progressive stage of MS, which are so far covered in experimental models only to a very limited degree. Thus, as summarized in Table 2, new experimental models have to be developed to address these questions.

**Table 2:** Future research strategies for experimental multiple sclerosis research

MS-related topic	MS pathology	Suggested strategy
Role of CD8^+^ T-cells	Major contribution of CD8^+^ T-cells in the inflammatory process of MS [24, 53, 62]	(1) Expand knowledge on mechanisms of inflammation and tissue injury in existing models of CD8^+^ T-cell-mediated brain inflammation (2) Define mechanisms, how CD8^+^ T-cell autoimmunity can be induced by active sensitization
Role of B-cells	Therapeutic effect of B-cell depleting therapies [61, 150, 152]	Create new in vivo models to test the role of B-cells in neuroinflammation
Mechanisms of Demyelination	Presence of a soluble demyelinating (cytotoxic) factor in serum and cerebrospinal fluid of MS patients [98]	(1) Define the nature of the demyelinating factor in serum and CSF of MS patients beyond anti-MOG antibodies (2) Define its role in different models of brain inflammation in vivo
Models for progressive MS	MS lesions develop on the background of pro-inflammatory microglia activation seen already in the normal white matter of age matched controls [53, 144]	(1) Test the effect of different microglia pre-activation in different models of brain inflammation (a) non-SPF environment (b) systemic innate immunity activation prior to induction of autoimmune inflammation (c) genetic models of microglia pre-activation
	Mitochondrial damage and “virtual” hypoxia play an important role in demyelination and neurodegeneration in MS, being most pronounced in the progressive stage [103, 104]	(1) Define mechanisms of tissue injury in mitochondrial mutants (2) Combine models of mitochondrial dysfunction with models of brain inflammation

In addition, strict rules of experimentation have to be followed to achieve definite and reproducible results. This appears to be highly important, since reproducibility of published experimental studies has sometimes been a problem, and many results from such studies have failed to show comparable effects, when tested in patients. Recently guidelines, which set the standard for publication and grant application, have been formulated for EAE research [6], but similar rules also apply for the other models described in this review.
